# Evolving Consciousness: Insights From Turing, and the Shaping of Experience

**DOI:** 10.3389/fnbeh.2020.598561

**Published:** 2020-11-12

**Authors:** Thurston Lacalli

**Affiliations:** Department of Biology, University of Victoria, Victoria, BC, Canada

**Keywords:** sentience, evolutionary innovation, qualia, hard problems, emergence, neural correlates of consciousness

## Abstract

A number of conceptual difficulties arise when considering the evolutionary origin of consciousness from the pre-conscious condition. There are parallels here with biological pattern formation, where, according to Alan Turing’s original formulation of the problem, the statistical properties of molecular-level processes serve as a source of incipient pattern. By analogy, the evolution of consciousness can be thought of as depending in part on a competition between alternative variants in the microstructure of synaptic networks and/or the activity patterns they generate, some of which then serve as neural correlates of consciousness (NCCs). Assuming that NCCs perform this function only if reliably ordered in a particular and precise way, Turing’s formulation provides a useful conceptual framework for thinking about how this is achieved developmentally, and how changes in neural structure might correlate with change at the level of conscious experience. The analysis is largely silent concerning the nature and ultimate source of conscious experience, but shows that achieving sentience is sufficient to begin the process by which evolution elaborates and shapes that first experience. By implication, much of what evolved consciousness achieves in adaptive terms can in principle be investigated irrespective of whether or not the ultimate source of real-time experience is known or understood. This includes the important issue of how precisely NCCs must be structured to ensure that each evokes a particular experience as opposed to any other. Some terminological issues are clarified, including that of “noise,” which here refers to the statistical variations in neural structure that arise during development, not to sensory noise as experienced in real time.

## Introduction

The literature on the subject of consciousness is vast and diverse, reflecting the range of interests of those who write on the subject, from philosophers to neuroscientists (Velmans, 2009; Van Gulick, 2018). With few exceptions (e.g., Barron and Klein, 2016; Friedman and Sovik, 2019), the perspective is usually top-down and human-centric, which, as Cisek (2019) has pointed out, tends to focus on questions that are not always meaningful from a strictly biological perspective. One especially under-investigated issue (Ginsburg and Jablonka, 2019) is the question of how, through the agency of natural selection, an innovation like consciousness can emerge in evolution, including whether it is easy or hard to evolve in its simplest manifestation, sentience. Such questions are relevant to this collection of articles because, if sentience (and hence, consciousness) is easy to evolve, it should occur wherever it confers a selective advantage, and so be distributed more widely across animal taxa than it is generally thought to be.

Among the classical hard problems of consciousness (Feinberg and Mallatt, 2016, for example, list four) the one generally considered the most fundamental and intractable is the question of how it is possible to have a subjective experience in the first place (Chalmers, 1995; Levine, 2009; Majeed, 2016). From an evolutionary perspective, one can assume the existence of some kind of precursor of subjective experience while, at the same time, recognizing that there is no way to prejudge what this is or whether it arises in a way that can be explained by known physical laws. The generation of even a rudimentary glimmer of subjective experience from any such precursor is then a signal achievement, as distinguishing this glimmer from its absence is as much an example of evolved consciousness as the more fully elaborated version familiar to members of our species. The present article is designed to investigate more fully how subjective experience first emerged in evolution, by providing a framework for dealing with some of the inherent conceptual problems. The framework is borrowed from the study of pattern formation, specifically the formulation devised by Turing (1952), which has parallels with the issue at hand.

The emergence of pattern during biological development is a situation where something, i.e., macroscopic pattern, emerges over time, apparently from nothing, i.e., the homogeneous un-patterned condition. Here the physical basis of what is happening is far closer to being understood than is the case for consciousness. It is instructive therefore to probe this example more deeply for the insights it may provide, which turn out to depend a good deal on appreciating the role played by the statistical behavior of discrete entities, molecules in the case of pattern, but something else when the analysis is applied to consciousness. Turing’s formulation nevertheless yields only a partial solution to the problem of consciousness because, while the emergence of particular structures and circuit dynamics can be dealt with, the emergence of real-time conscious experience from the pre-conscious condition is beyond its remit. This accords both with philosophical doubts as to whether any reductive explanation will be found for such questions (Chalmers, 1995), and with the distinction drawn between weak and strong emergence (Bedau, 1997; Kim, 2016). According to Bedau, dynamical models like Turing’s can explain only examples of the former, but not the latter, for which the properties of the emergent higher-level domain, consciousness, in this case, are not deducible from those of the lower-level domain, neural structure and activity. It is nevertheless useful to identify the reasons for this failure in analytical (i.e., mathematical) terms because there are multiple forms of emergence to consider, of the phenomenon of consciousness itself, and its structural and functional correlates, both during development and through evolutionary time. There are many opportunities here for confusion, as to what precisely is emerging, and from what, and whether a given evolutionary innovation represents an example of emergence or not, and of what kind.

Besides emergence, Turing’s formulation is useful also when thinking about the precision and reliability of developmental outcomes. The Turing mechanism is relevant here because of its ability to act as an amplifier, extracting a signal from a noisy background, thereby reducing the errors that inevitably arise in the noisy real world of molecular and cellular processes (Holloway and Harrison, 1999; Rao et al., 2002; Balázsi et al., 2011). With regard to consciousness, the question one then wants to address is how tightly controlled neural structure and patterns of activity must be to ensure that a particular experience, rather than some other, is evoked due to the action of a given neural correlate of consciousness (NCC). Knowing the answer will be important if and when it becomes possible to identify and study NCCs directly, because the prevalence of mechanisms for error correction during their development will reflect how precisely NCCs must be structured to perform their allotted task, while providing, at the same time, a measure of how sensitive conscious experience is likely to be to incremental change at the genomic level.

Specific features of real brains are not considered in this account, nor questions concerning when, or where in the brain, vertebrate consciousness first originated, an intentional omission given that the focus here is on issues that apply irrespective of taxon. A brief discussion is included regarding the role of relational ideas for solving the hard problems of consciousness (strong emergence in this context), where “relational” is taken to refer to formulations that, in contrast to those explored here, are neither developmental, evolutionary, nor coordinate-dependent.

## Evolutionary Conundrums

Several conceptual problems arise when explaining how anything truly novel arises in evolution (Moczek, 2008; Shubin et al., 2009), but typically rudiments or precursors of some kind can be identified, whether at the molecular, cellular, or anatomical level, on which evolution acts to produce the innovation in question. Take the example of an eye assembled by evolution from a set of preexisting parts, including photoreceptors, neurons, and pigment cells. At some point in the sequence, there would be a transition from an anatomical feature that is not recognizable to us as an “eye” to one that is. This distinction does not matter to evolution, however, which is concerned only with the benefits to survival and reproductive success that result from improved visual capabilities. So the concept of an “eye” in this case, as a particular arrangement of parts, is largely a matter of semantic convenience. And, because evolution in this case involves a reordering of parts that were already present, the innovation is largely in the reordering rather than the parts themselves. Does this apply also to consciousness? The argument developed here is that it does, which makes the problem a less daunting one, of understanding precisely what we mean by “parts,” and clarifying, based on physical principles, how these can be suitably ordered to evoke a conscious experience in a consistent and reliable way.

Since we have no clear idea of the physical processes ultimately responsible for conscious experience, defining the “parts” just referred to is a matter of some debate. It should already be evident from the Introduction that my approach to the problem is reductionist and focused on NCCs, on the assumption (e.g., as expressed by Mallatt and Feinberg, 2017) that consciousness must depend in some way on specific features of neural structure and function. In practical terms, what I am seeking is a guide to how NCCs might be identified, given that there is a reasonable prospect of eventually obtaining essentially complete datasets on the neural circuitry and functional properties of brain tissue from model organisms ranging from flies to vertebrates (e.g., Marques et al., 2019; Yu et al., 2019). As to controversies regarding how NCCs are best defined (Chalmers, 2000; Fink, 2016), or what their role as causative agents may be (Hohwy and Bayne, 2015; Polak and Marvan, 2018), the key point is that for hypothetical NCCs, as here, a degree of causality stronger than simple correlation can be assumed if we single out those NCCs or ensembles of NCCs that together are the proximate cause (Polak and Marvan’s regular cause) that a particular experience is evoked as opposed to any other experience. This assists the construction of thought experiments framed in topological terms (as below, in the section “The Turing Mechanism, part 2”), where the mapping is assumed between the physical realm of neural structure and function and an abstract experience space consisting of all possible experiences.

## The Turing Mechanism, Part 1: Order From Fluctuations

Turing (1952) was the first to show mathematically that patterns could emerge from an initially homogeneous chemical system consisting of two mutually interacting and diffusing reactants. His analysis has since been applied to various examples of biological pattern and, though some of these are now known to arise *via* mechanisms other than Turing’s, his proposal has had its share of successes (e.g., Maini et al., 2006; Kondo and Miura, 2010; Green and Sharpe, 2015), and there is now a sub-discipline of chemistry that deals with chemical systems showing similar behavior (Grzybowski, 2009). Turing’s mechanism is a hypothetical construct, i.e., an idealized set of reactions, and only represents in a general way the kinds of interactions expected of real molecules in a biological setting. It still has considerable heuristic value, along with its close theoretical kin, the Brusselator and the Gierer-Meinhardt model (Harrison, 1987), for showing how a pattern can arise spontaneously in a predictable way.

**Figure 1:**
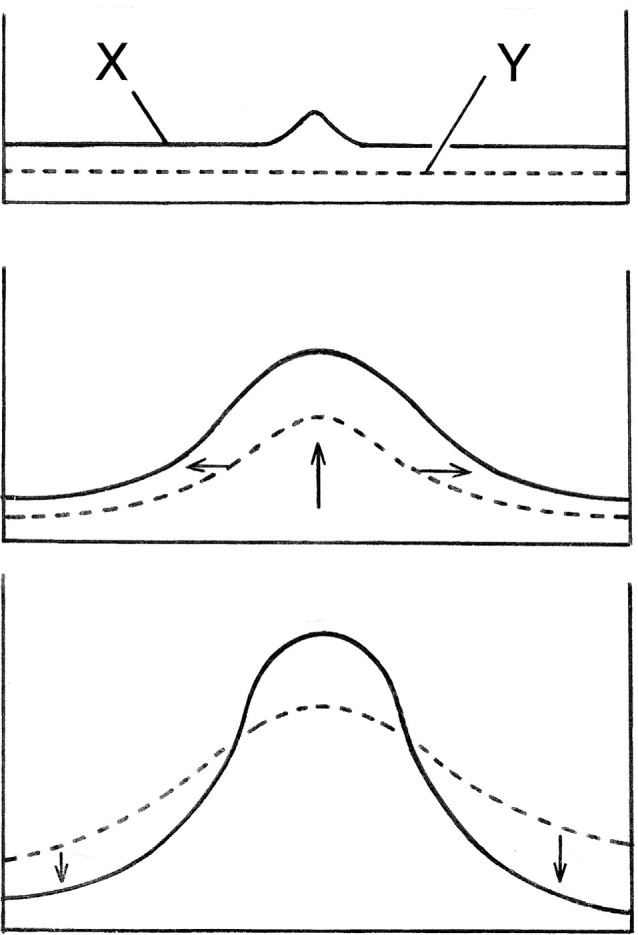
A simple explanation of how Turing’s mechanism generates a spatial pattern, here shown as concentrations peaks for two diffusing substances, X and Y, which interact such that X stimulates its own production and that of Y, while Y inhibits X production. The homogeneous steady state is maintained by a balance between supply and degradation of both substances, so this is an open, dissipative system, and with appropriate parameter values, the steady-state is unstable. A random deviation in X from the steady-state will then grow (top frame), which stimulates extra Y formation, so Y grows as well (middle frame, vertical arrow). Y diffuses more rapidly (horizontal arrows), and so spreads laterally and drives down X (bottom frame), thus stabilizing the central peak in X. This provides an intuitive understanding of the mechanism works, but largely conceals the crucial role played by statistical behavior at the molecular level.

There are various ways of explaining how the Turing mechanism does this. The most easily comprehensible, popularized by Maynard Smith (1968), shows how, at a macroscopic scale, a small deviation from the homogeneous steady state can grow and develop into something more substantial (Figure 1). This occurs if the slower diffusing of the two components required, usually called the activator (X in the figure), enhances both its own production and that of a faster diffusing second component (Y), which then inhibits the surrounding region from producing additional activator peaks. This gives some insight into why the un-patterned initial conditions are unstable, but the question of where the pattern actually “comes from,” requires a more thorough examination of the solutions to Turing’s equations and the physicochemical reality they embody.

Harrison (1993) devotes considerable space to both of these topics, with a focus on understanding the statistical nature of thermal fluctuations in a molecular system, of say, a solute in a solution. At a macroscopic level, the solution is homogeneous, but this conceals, at the molecular level, the constant motion of the solute and solvent molecules as they jostle back and forth. In mathematical terms, these fluctuations can be represented in the linear limit as a harmonic series, i.e., a sum of sines and cosines, representing the contribution of components of different spatial scales (wavelength) to the whole. As fluctuations occur, being local, they contribute amplitude to the short-wavelength components of the sum, but these are fleeting and decay rapidly. Long-wavelength components will be present as well, even if minuscule for any one fluctuation, but they dominate over time. The statistical view of homogeneity is then of a sea of fluctuations arising and disappearing at the microscopic level, with homogeneity being sustained at the macroscopic level because, under normal conditions, the long-wavelength components always dominate.

Under unusual conditions, of which Turing’s mechanism is one, a balance of reaction processes and diffusion can act to selectively amplify certain of the shorter wavelength components so that a pattern of fixed size emerges. If we then ask where the pattern came from, the answer is that it was already there, hidden in the shorter wavelength components of the fluctuations. Without a suitable amplifier, however, these never manifest themselves at a macroscopic scale. So the pattern in a sense is both “there” and “not there” simultaneously, which is otherwise at odds with ordinary experience. Conceptual biases inherent in the latter can be difficult to overcome, as Boltzmann found when he first introduced a statistical way of dealing with events at the molecular scale (Cercignani, 1998), but statistical mechanics has now been, for a century, the accepted way of dealing with such processes. For a more detailed treatment of the subject, Nicolis and Prigogine (1977) can be consulted, but Harrison’s account (Harrison, 1993) is more accessible for the non-specialist (see especially chapters 5 and 7).

## The Turing Mechanism, Part 2: A Device for Shaping Experience

The reactions represented in Turing’s equations are idealized, but the mathematics, and the physics it represents, are well accepted and precise, as is the form of the solutions. The same cannot be said if we try applying his analysis to consciousness, because there the physical processes we suppose to be most relevant range from being imprecisely known to entirely hypothetical, and there is no body of accepted mathematical theory to guide us. The argument developed here therefore depends on numerous assumptions, and at best represents an approximation of reality. Even within these limits, however, there are useful insights to be gained from the exercise.

**Figure 2:**
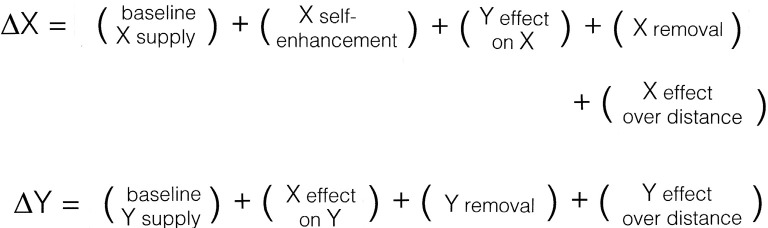
The components needed by Turing’s mechanism to form patterns, expressed formally here as changes over time (the deltas) of two variables (X and Y) depending on their supply, removal, and the interactions in which they participate. One then has to find an appropriate way for each of these processes to be expressed mathematically, and some of the plus signs will (indeed, must) become minus signs. In this analysis, the model is taken to represent a developmental process, with the Xs and Ys interpreted as structural in nature (see text for details), so the emergence of a “pattern” reflects changes to the way neural circuitry is locally ordered in three-dimensional space during brain development. Consciousness enters only because of the way the variables are interpreted, in this case by supposing that X-dependent synaptic reordering affects subjective experience in some way, but without specifying how. The model can then be used to address questions about the emergence during development of structural features capable of evoking or otherwise affecting conscious experience, but says nothing about the nature or origin of experience itself.

Consider first what the Xs and Ys of the mechanism (Figure 2) might be. If our focus is to be on NCCs, then it is reasonable for X and Y to be participants in the developmental process by which a particular circuitry or feature of neural organization is produced. Having only two such variables is an oversimplification, because the development of even moderately complex neural circuits would depend on many such Xs and Ys, involving multiple cell types and their myriad synaptic and non-synaptic interactions. The simplest case serves only as an illustration of how, as a first approximation, the variables might be defined. They could equally well be activity-based, to reflect the functional properties of developing circuits, but structural variables fit more easily in Turing’s model in its original form, requiring fewer additional assumptions, and the outcome of the analysis is not, in any case, changed, at least with regard to the advantages and limitations of this type of formulation.

So, for the sake of argument, take X to be a measure of a constraint on the space available for synapse formation along the dendrites of a particular set of neurons, providing sites, ordered in a particular way, that are either filled or not filled by synapses from the branched terminals of presynaptic fibers represented by Y. An XY interaction would either initiate synapse formation, reducing X at the expense of Y, or not, meaning the reverse, which also then reinforces the X-dependent ordering effect. This produces an inverse relation between X and Y, which is required for Turing’s mechanism to generate patterns. Note, however, that X and Y no longer represent concentrations of chemical species, but are quantitative measures of some feature of larger-than-molecular scale. Hence there is a spatial dimension to the XY interaction that must be reflected either in the mathematical expressions chosen or the way they are interpreted.

One could ask why a Turing mechanism is needed here at all, as the XY interactions will inevitably produce a synaptic structure of some kind, and this can be adjusted incrementally over time as natural selection acts to alter the genes on which the Xs and Ys depend. To restrict the analysis just to changes at the genomic level, however, assumes that only jigsaw-like self-assembly processes need to be considered, as in the assembly of a virus particle (Harrison, 1987). While neural development will likely be more rigidly controlled in some animals than others (e.g., in flies more than mice), there are potential sources of variability at multiple levels in neurogenesis in any animal, in the precise positioning of cell bodies, dendritic arbors, synapses along those arbors, and many other features. How ubiquitous this variability is, has become evident to me from my work on amphioxus (e.g., Zieger et al., 2017), a very simple system, yet one showing a remarkable degree of opportunism in the way neuronal contacts are established, requiring a compensatory redundancy in neuronal specification and positioning, even when the interactions involve only a few cells and cell types. Given the vastly greater complexity of the brain centers associated with consciousness, in chordates at least, it is difficult to imagine how the circuits involved can be assembled correctly without the intervention of mechanisms specifically evolved to reduce variability of outcome. The Turing mechanism is only one possible way to do this, but illustrates in an especially clear way how the competitive dynamics of the assembly process can be harnessed to favor some outcomes over others. Hence the pattern that emerges is not simply a structured set of synapses, as some kind of structure will inevitably be produced, but a particular structure rather than some other, and one that can be precisely replicated in each generation.

The Turing mechanism is a dynamic process where the entities represented by the variables, in this case, the sites available for synapse formation and the developing terminals, are continually supplied and removed. This is reflected in the inclusion of supply and removal terms in Figure 2. As to the XY interactions, the analogy implies that these should be thought of as resembling collisions between chemical reactants, that is, repeated contact events of which only a fraction become stable synapses. For the patterning process to unfold, a certain amount of developmental time is required for ordered structure to emerge, or more precisely, given the way X and Y are defined, for a structure to emerge that is ordered in a different way than would otherwise have been the case. The initial conditions can both bias and expedite this process, the large initial fluctuation in Figure 1 being an example, as it gives a significant head start to a subset of possible patterns. For a tissue domain that is already heterogeneous because of underlying gradients and local signaling centers, patterning would proceed at a much faster rate.

Autocatalysis is a crucial feature of Turing-type mechanisms, represented here by the X-enhancement step. So, having more X generates yet more X at a greater than linear rate. Finding a realistic way to represent this in mathematical form has proven problematic for many Turing-type models, as it is here, and typically requires terms in higher powers of X (e.g., *X*^2^) for which there may be limited mechanistic justification. For the present case, one could perhaps think of such terms as arising from spatial effects, so that, for example, if the dynamics involved dendrites that branch and produce spines, each with multiple sites for synapse formation, there could be alternative ways of configuring the branches such that having more sites available (i.e., more X) promotes the formation nearby of yet more sites of the same type (even more X) at a greater than linear rate. Or, activity-based mechanisms might be acting to sustain synaptic connections, comparable to the role played by long-term potentiation in learning and memory (Lynch, 2004), but operative during development, as indeed they do in some instances (Rauschecker, 1991; Cruikshank and Weinberger, 1996). So, for example, X might grow at the expense of Y due to competition between Y and non-Y synapses, where the latter are stabilized preferentially by potentiation, reducing the number of sites that Y can occupy and affecting the way “X sites not occupied by Y” are spatially distributed. The Turing mechanism would then be a hybrid model, combining structure and circuit dynamics, both of which would contribute to the synaptic reordering. The variables themselves could still be defined in exclusively structural terms, as here, or could be redefined to incorporate activity-based features explicitly.

In addition to the above requirements, both X and Y must have effects that propagate over a greater distance, comparable to the diffusion terms in Turing’s equations. Perhaps a failure to form synapses at one site would cause sprouting nearby and more Y synapse formation there, hence giving a kind of mobility to Y comparable to a diffusional effect. For X, assuming increasing X might affect the way dendritic arbors are locally configured, there would be a knock-on effect at a moderate distance (e.g., on adjacent arbors) that is less than that over which Y acts, giving Y the greater effective mobility, as required by the mechanism.

The precise identities of X and Y and the nature of the XY interaction are less important here than the general point, that in the development of real brains there are potentially many opportunities for X-type structural and activity-based variables to respond to Y-type synaptic inputs in ways that produce dynamics of the kind needed by a Turing mechanism. A preliminary investigation of ways to express this mathematically, adapted from Lacalli and Harrison (1979), has yielded provisional results showing that, for a suitable choice of reaction terms and parameter values, models based on Figure 2 can form patterns. So in this formulation, a spatially restricted domain could emerge within the brain where X and Y have diverged quantitatively from the un-patterned condition, resulting in a local reordering of the synaptic organization or, for an effective wavelength that is small relative to the area being patterned, an ordered array of such domains. If we then assume this reordering correlates with consciousness in some way, what has emerged is, by definition, an NCC. Note, however, that neither consciousness nor, indeed, anything relating to subjective experience appears explicitly in this formulation, but enters only because of the interpretation we choose to place on the terms in the equations, in this case, that “X-dependent synaptic reordering” contributes to consciousness. Because the formulation says nothing about how this happens, emergence in this instance is weak rather than strong.

Now consider the evolutionary part of the story: evolution enters through its ability to alter the Xs and Ys themselves, increasing the frequency in the population of those X and Y variants that, acting through the effect they have on the emerging consciousness, most benefit survival and reproductive success. The appropriate way to explore this aspect of the problem is through Directionality Theory (Demetrius, 2013; Demetrius and Gundlach, 2014), which deals statistically with changing gene frequencies over evolutionary time, i.e., from generation to generation. But here also there is no way to introduce conscious experience explicitly, first because the equations are concerned only with outcomes, on survival, reproductive success, and gene frequencies, but also because of the incompatible time scales, real time for consciousness vs. evolutionary time for evolution.

In consequence, there is no obvious route to incorporate anything explicitly related to consciousness as a phenomenon into either the developmental or evolutionary part of the analysis, either as to what consciousness “is,” how it is experienced in real time, or what its precursor might have been in the preconscious brain. Perhaps the argument could be recast in a more revealing way, but on the evidence available, it appears that an analytical treatment of the evolutionary process, whether combined with development or not, is inherently limited in what it can say about the nature of conscious experience. This accords both with philosophical argument, by Chalmers (1995) for example, and our conception of how natural selection operates: that just as it does not matter to evolution that it has produced an “eye,” only that visual performance has been improved in ways that enhance survival and reproductive success, neither should evolution be concerned with what consciousness “is,” only that it is useful. From this, it is possible to give a spare but quite precise definition of what consciousness is “for,” meaning its function when viewed from an evolutionary perspective: that it is a mechanism for restructuring synaptic networks in ways that would not otherwise have occurred, to produce advantageous behavioral outcomes that would not otherwise have happened.

On a more positive note, the patterning analogy provides a framework for thinking about how conscious experience can be shaped by development and evolution acting in concert. Among the “parts” being shaped is the precursor mentioned in the Introduction, which differs in character from those “parts” based in structure and circuitry dynamics, i.e., the Xs and Ys. What we therefore learn about emergence from this exercise is the importance of paying attention to precisely what is emerging, and from what, a point taken up in the next section with regards to the concept of “noise.”

To conclude this section, it is useful to again emphasize the role mechanisms like Turing’s can play in ensuring reliable outcomes during development, e.g., that a hand adapted to have five digits reliably does so, as opposed to having a statistical scatter of, say, 3–8. In this respect, Turing’s mechanism is doing two jobs at once: generating pattern from homogeneity, while also ensuring that the pattern is a particular one, e.g., of stripes rather than spots, or a fixed number of discrete structures. In doing so, it acts as an error-reduction mechanism, taking in inherently noisy input, and converting it into a repeatable outcome that overcomes the randomizing tendency inherent in real developmental processes, whether at the molecular, subcellular, or cellular level.

This then provides a way of assessing the importance of precision of outcome to any developmental process under investigation because, if a high degree of precision is required, evolution will have incorporated the necessary corrective mechanisms. For NCCs, there is a useful topological way to think about the consequences, as follows: consider the mapping between an abstract neural structure space consisting of all possible NCCs and an experience space consisting of all possible experiences. If NCCs are not required to be especially precise in order to evoke a particular conscious experience, then many points in neural structure space, representing numerous NCC variants, will map to the same point in experience space. If great precision is required, then correspondingly fewer NCC variants will map to any one such point. The map then represents in a formal way how precisely conscious experience depends on events in the physical world, but also how experience is shaped by evolution since, if even small changes to NCC configurations change experience, then that experience can be easily altered by incremental change at the genomic level. Conversely, for maps with greater redundancy, where many NCC variants map to the same point in experience space, a degree of evolutionary inertia would be predicted, since many changes to the NCCs must accumulate before their effect is seen in experience space. Whether this can be used to make meaningful predictions about the absolute rate at which conscious experience can evolve remains to be determined, but is a possibility worth considering.

## Understanding “Noise” and the Search for NCCs

Since pattern emerges in Turing’s mechanism from rudiments of pattern that are already present in the homogeneous state, i.e., the fluctuations, one could ask whether, by analogy, this means that all the elements needed to construct consciousness are already present before consciousness evolved, needing only to be selectively amplified and reordered. In a general way, this can be thought of, as with pattern formation, as consciousness emerging as a signal from a background of noise, but with an important caveat as to the meaning of the term “noise.” For noise as a real-time subjective experience, a plausible model would be the buzz of static from a radio tuned to no particular station, i.e., something random and without a meaningful signal. Perhaps this is what neural activity in animals without consciousness produces, but if so, we are left with a somewhat unsatisfactory situation of accounting for the origin of real-time subjective experience by assuming it is there from the start. The solution to this conundrum is to recognize that when the variables relate to structural features, the corresponding “noise” relates only to variations in structure, and not to the quality of the experience, noise-like or otherwise.

In consequence, the patterning analogy is silent on the issue of whether animals without consciousness have, or do not have, anything resembling subjective experience. This then resolves any confusion over whether all possible experiences are already present in the preconscious condition as consciousness began to evolve, in an analogous way to the presence of all possible wavelengths in thermal fluctuations as pattern forms. Here I believe the analogy leads to a sound conclusion, that indeed rudiments of all possible experience could already be present in the preconscious state. But the “rudiments” here are not rudimentary experiences, but rudimentary assemblages, at the organizational and micro-circuitry level, of the building blocks needed for consciousness to evolve over a series of subsequent generations. Like LEGO scattered across the floor, they await assembly in order to become something specific.

The issue of how real-time noise might be experienced by an individual brain is nevertheless worth considering further, for what it says about the nature and localization of NCCs. The point here is that one cannot assume *a priori* that animals lacking evolved consciousness also lack any kind of subjective experience. If they did in fact experience a buzz of random real-time noise as mentioned above, then the emergence of a meaningful signal from this during evolution, localized to one part of the brain, would necessarily have to be accompanied by the suppression elsewhere of any real-time noise that might compete with that signal. A search for NCCs would then reveal two kinds, one correlated with specific experiences, the other for suppressing noise. Evidence of NCC-related restructuring would, in consequence, be widespread in the brain, which could complicate the problem of determining from synaptic microstructure where the NCCs for a given subjective experience were localized. The analysis presented here is entirely agnostic on this issue, so neither alternative can be ruled out: that consciousness emerges in evolution incrementally from a sea of real-time noise, or from a background of subjective silence.

## Discussion: Caveats, Hard Problems, and the Relational Stance

A virtue of the approach taken here, and perhaps its most important result, is to show how the rather daunting problem of investigating the origin of consciousness can be simplified by separating the developmental and evolutionary components from the vast array of theories and hypotheses devised to account for consciousness as a phenomenon. The elaboration and refinement of subjective experience can then be dealt with in a straightforward way, as the result of routine developmental and evolutionary processes, while more problematic issues, chiefly those concerning the nature and origin of conscious experience, can be deferred. A key question is then whether the failure to address the latter is specific to this analysis, or is general to any evolutionary formulation. The question is important because, if natural selection is truly agnostic regarding the nature of the real-time precursors on which it acts, it follows that once the first sentient experience has emerged in a taxon, evolution can complete on its own, through its ability to shape experience, the process of converting that first experience to fully evolved consciousness in all its baroque complexity.

A further problem, not addressed in the above analysis, is that for natural selection to act at all to shape experience, and hence for consciousness to evolve over time, there must be a route by which emergent experience can influence the real world through its action on behavior. Yet behavior is fully under the control of neurons, and as conscious experience first began to emerge in evolution, it is non-trivial to account for how it could be anything other than a by-product of neural activity, i.e., an epiphenomenon, incapable of altering behavior in and of itself. This conundrum remains a contentious issue among philosophers (Gadenne, 2006), but if the emergence of consciousness is to be explained as a product of natural selection, a link between experience and behavior appears to be unavoidable (Popper and Eccles, 1977, part I). I raise this issue to point out the consequences for NCCs, which have so far been discussed only in relation to the refining and shaping of experience. It is a separate question whether the first emergence of a link connecting experience to behavior is embodied in these same NCCs or requires a separate set of neural pathways. Because behavioral responses involve effector pathways as well as sensory ones, one could argue that, whereas refining experience could be accomplished by neurons acting in concert in small-scale local circuits, the link between emerging conscious experience and behavior might instead depend on non-local pathways linking multiple brain regions. This is, however, no more than conjecture.

A final issue concerns whether any mechanism involving material entities embedded in three-dimensional space can say anything useful about a phenomenon that is neither material in nature nor assignable to a specific spatial location. If the answer is “no,” then we are left by default to address the hard problems of consciousness using formulations that are relational in character, and hence neither structure- nor coordinate-dependent. There are many examples of relational analysis across the sciences. In physics, for example, it figures prominently in quantum mechanics and investigations into gravity and the nature of space-time (e.g., Anderson, 1964; Smolin, 2006). For theories of consciousness, however, the relation more usually arises from the network structure of a neural substrate that processes either information in the abstract, or neural signals in more concrete formulations, using mammalian cortex and cortico-thalamic circuits (e.g., see Butler, 2008; Ward, 2011) and our own conscious experience as models. This would include global workspace models (Dehaene and Naccache, 2001; Dehaene, 2014), variants of integrated information theory (Oizumi et al., 2014), and much of what falls in the category of higher-order theories (Brown et al., 2019; Carruthers and Gennaro, 2020) and computational theories of cognition (Piccinini and Bahar, 2013). Such models typically deal with consciousness as a single phenomenon, complete with all the complexities familiar to members of our species. But there is no *a priori* reason to suppose that human experience is a good model for consciousness as it first emerged in evolution, or that a certain level of structural, integrative, or computational complexity, in and of itself, is a necessary or sufficient condition either for sentience or more highly evolved forms of consciousness (Manzotti, 2013; Wood, 2019).

A better starting point might be to devise a relational theory potentially applicable to a wider range of brains, both vertebrate and invertebrate, regardless of whether they possess anything organizationally comparable to the mammalian cortex. One proposal that is less problematic in this regard, by Merker (2013), attributes consciousness to a more abstract kind of relation, between a “self” and the “sensory representations” of the external world to which the self is the witness. It is difficult to cast this conception in more concrete terms, but fewer assumptions are required concerning the neural substrate that implements the relation, aligning this proposal more closely with my conclusions regarding the hard problems: that solving them for the very first glimmerings of sentience solves them fully. If sentience in vertebrates predates mammals or has evolved independently in other lineages, then cortico-thalamic pathways are neither the only, nor perhaps even the best place to look for the circuitry innovations, and hence the NCCs, that make consciousness possible.

## Author Contributions

TL is solely responsible for the preparation and content of this article.

## Conflict of Interest

The author declares that the research was conducted in the absence of any commercial or financial relationships that could be construed as a potential conflict of interest.
